# α-Catulin promotes cancer stemness by antagonizing WWP1-mediated KLF5 degradation in lung cancer

**DOI:** 10.7150/thno.63627

**Published:** 2022-01-01

**Authors:** Chia-Hao Tung, Meng-Fan Huang, Chen-Hsien Liang, Yi-Ying Wu, Jia-En Wu, Cheng-Lung Hsu, Yuh-Ling Chen, Tse-Ming Hong

**Affiliations:** 1 Institute of Clinical Medicine, College of Medicine, National Cheng Kung University, Tainan, Taiwan.; 2 Institute of Basic Medical Sciences, College of Medicine, National Cheng Kung University, Tainan, Taiwan.; 3 Clinical Medicine Research Center, National Cheng Kung University Hospital, College of Medicine, National Cheng Kung University, Tainan, Taiwan.; 4 Division of Hematology-Oncology, Department of Internal Medicine, Chang Gung Memorial Hospital, Chang Gung University, Taoyuan, Taiwan.; 5 Institute of Oral Medicine, College of Medicine, National Cheng Kung University, Tainan, Taiwan.

**Keywords:** α-Catulin, cancer stem cell, KLF5, WWP1, non-small cell lung cancer.

## Abstract

**Background:** The cytoskeletal linker protein α-Catulin has been shown to be important for tumor progression in various cancers. However, its role in the regulation of cancer stemness remains unclear.

**Methods:** Phenotypic effects of α-Catulin on the cancer stem cell (CSC)-like properties and metastasis were examined by *in vitro* sphere formation assay, migration assay, invasion assay, and* in vivo* xenografted animal models. Yeast two-hybrid assay, co-immunoprecipitation assay, and cycloheximide chase assay were performed to confirm the effect of α-Catulin on the WWP1-mediated degradation of KLF5. CPTAC and TCGA database were analyzed to determine the clinical association of α-Catulin, KLF5, and stemness-associated signatures in lung adenocarcinoma.

**Results:** We report that α-Catulin increases cancer stem-like properties in non-small cell lung cancer (NSCLC). The expression of α-Catulin is elevated in tumor spheres compared to sphere-derived adherent cells and promotes the acquisition of cancer stemness characteristics *in vitro* and *in vivo*. Mechanistically, the interaction of α-Catulin and the C-terminal region of Kruppel-like transcription factor KLF5 results in the inhibition of WWP1-mediated degradation of KLF5. Accordingly, increased protein expression of KLF5 is observed in clinical specimens of lung adenocarcinoma with high expression of α-Catulin compared to specimens with low α-Catulin-expression. Knockdown of KLF5 abrogates α-Catulin-driven cancer stemness. α-Catulin is known to interact with integrin-linked kinase (ILK). Notably, an ILK inhibitor disrupts the α-Catulin-KLF5 interaction, promotes the degradation of KLF5, and decreases α-Catulin-driven cancer stemness. Importantly, we identify a *CTNNAL1*/*ILK*/*KLF5* three-gene signature for predicting poor overall survival in patients with lung adenocarcinoma.

**Conclusions:** These findings reveal a molecular basis of α-Catulin-enhanced KLF5 signaling and highlight a role for α-Catulin in promoting cancer stemness.

## Introduction

Lung cancer is the leading cause of cancer death in developed countries 1. Non-small cell lung cancer (NSCLC) accounts for more than 85% of all lung cancer cases 2. Most deaths of NSCLC patients are due to cancer metastasis and therapy resistance 3-5. Recently, mounting evidence suggests that cancer metastasis and therapy resistance are mediated by cancer stem cells (CSCs) 3. Lung CSCs have been identified by surface marker expression, sphere formation and side population detection 6. CSCs constitute a very small population within the bulk of the cancer cell fraction and share some similar functional properties with normal stem cells 6. For example, similar to stem cells, CSCs have high differentiation potential, show infinite proliferation and self-renewal capacity and play key roles in tumor growth and metastasis 6. In the clinic, CSCs are known to be resistant to chemotherapy and radiotherapy 6, 7. Some reports have shown that CSCs have high tumorigenicity capacity and are protected by multiple resistance mechanisms, leading to tumor metastasis after treatment with radiation and chemotherapy 3, 6, 8. Therefore, targeting CSCs and understanding the molecular mechanisms that maintain CSC growth may reveal an efficient strategy for the treatment of human cancers.

α-Catulin, an α-catenin-related protein, is an oncoprotein that plays a role in the epithelial-mesenchymal transition (EMT) and cancer metastasis in many cancer types 9-12. High expression levels of α-Catulin have been observed in malignant melanoma cells compared to normal human melanocytes 9. α-Catulin has been shown to play a critical role in melanoma progression, triggering the epithelial-mesenchymal transition by suppressing the expression of E-cadherin and promoting the expression of mesenchymal genes such as N-cadherin and Snail/Slug 9. Recent evidence has shown that α-Catulin plays important roles in inflammation, apoptosis and cytoskeletal reorganization. As a cytoskeletal linker protein, α-Catulin integrates and regulates the crosstalk between the nuclear factor-kappa B and Rho signaling pathways, promotes cell migration and increases resistance to apoptosis 13. In addition, it has been shown that α-Catulin contributes to cisplatin resistance by activating NF-κB, AP-1 and ERK phosphorylation in malignant melanoma cells 14. We previously reported that α-Catulin is highly expressed in clinical oral squamous cell carcinoma tissues and promotes tumor growth by preventing cellular senescence 15. α-Catulin has also been found to be a potential tumorigenic factor that directly interacts with IKK-β and activated nuclear factor kappa B (NF-κB) signaling, which promotes cancer cell migration and resistance to apoptosis 14. Despite great efforts to study α-Catulin, the role of α-Catulin in lung cancer stemness remains unclear.

Kruppel-like factor 5 (KLF5) is a member of the Kruppel-like transcription factor family. It has been shown to regulate many cellular functions, such as proliferation, apoptosis and development 16. As a DNA-binding transcription factor, KLF5 has been shown in previous studies to promote cancer cell proliferation and apoptosis resistance in different human cancers 16. Evidence has indicated that KLF5 also plays an important role in stem cell development and CSC regulation 17. KLF5 is involved in the self-renewal of mouse embryonic stem cells (ESCs) by mediating the activity of the ESC-like transcription factors Oct3/4 and Nanog, which also play important roles in CSC formation 18. In addition, KLF5 has been shown to enhance the CD44/CD133 cell population and plays an important role in the regulation of CSCs in hepatocellular carcinoma 19. It is now accepted that KLF5 is a short-lived protein that can be rapidly degraded by the ubiquitin-proteasome system in cancer cells 20. Although it has been reported that several mechanisms controlling the stability of KLF5 21, the involvements of α-Catulin in the stabilization of KLF5 remains unclear.

In our previous study, demonstrated a role for α-Catulin in promoting metastasis of lung cancer 12. In the current study, we found the induction of the EMT phenotype upon α-Catulin overexpression. There have been other reports of strong connections between the EMT and CSCs in several malignancies 22. Furthermore, our microarray analysis showed that α-Catulin overexpression increased the levels of several stemness-associated genes and signatures. Given that the role of α-Catulin in cancer stemness remains unknown, our goal was to elucidate the functional role and molecular mechanisms of α-Catulin in the regulation of lung cancer stemness. Moreover, we investigated whether targeting the α-Catulin-associated axis provides an effective therapy for suppressing lung CSCs.

## Materials and Methods

### Cell cultures and reagents

CL1-0 and CL1-5 NSCLC cell lines were cultured in RPMI 1640 medium (Invitrogen) supplemented with 10% fetal bovine serum (FBS; Gibco) and antibiotics. A549 cells were cultured in high-glucose DMEM medium (Invitrogen) supplemented with 10% FBS and antibiotics. HOP-62 cells were purchased from the National Cancer Institute and cultured in RPMI 1640 medium supplemented with 10% FBS, 2 mmol/L L-glutamine and antibiotics. All of the cell lines were incubated at 37°C in a humidified atmosphere with 5% CO_2_. ATCC cell lines were authenticated via short tandem repeat (STR) analysis, and all cell lines tested free of mycoplasma contamination. Reagents including cycloheximide (Sigma-Aldrich), MG132 (Sigma-Aldrich), and the ILK inhibitor OSU-T315 (MedChemExpress, HY‐18676) were used in experiments. Paclitaxel was kindly supplied by Dr. Keng-Fu Hsu (Department of Obstetrics and Gynecology, National Cheng Kung University, Taiwan).

### Gene set enrichment analysis (GSEA)

To investigate the α-Catulin-activated signatures in NSCLC cells, we downloaded the publicly available GSE40141 dataset from the Gene Expression Omnibus (GEO) database. GSEA was conducted with 10000 phenotype permutations using the *R/Bioconductor* package fgsea (version 1.16.0), ranking genes according to the log_2_-fold change (A549-pLKO-α-Catulin vs. A549-pLKO expression). To determine the *CTNNAL1*-associated signatures, we downloaded publicly available data on lung adenocarcinoma (LUAD) from The Cancer Genome Atlas (TCGA) database (TCGA-LUAD) using the University of California Santa Cruz (UCSC) Xena browser (https://xenabrowser.net/datapages/). The GDC HTSeq FPKM RNA-seq dataset (version 07-20-2019) was downloaded for analysis. A GSEA was conducted to determine Pearson's correlation coefficient. All gene sets were obtained from the Molecular Signatures Database (MSigDB, http://software.broadinstitute.org/gsea/msigdb/) and Miranda's study 23. Gene sets with a false discovery rate (FDR) < 0.05 were considered significant.

### Western blot analysis

Protein lysate was subjected to SDS-PAGE and transferred to a PVDF membrane (0.45 µm, Millipore). Protein expression was analyzed by Western blotting with primary antibodies and horseradish peroxidase-conjugated secondary antibodies. Specific proteins were detected using Western Lightning™ Chemiluminescence Reagent Plus (PerkinElmer, NEL105001EA). The primary antibodies used for Western blotting were anti-α-Catulin (Abnova, H00008727-B01P), anti-KLF5 (Millipore, 07-1580), anti-FLAG M2 (Sigma-Aldrich, F1804), anti-c-Myc tag (Millipore, 05-419), anti-β-actin (Sigma-Aldrich, A5441), and anti-Lamin B1 (Abcam, ab16048).

### Lentivirus generation and infection

For overexpression of α-Catulin, α-Catulin cDNA was generated and cloned into a pLKO-AS2.neo lentiviral vector. Following transfection of this plasmid into HEK293T packaging cells, viral supernatants were collected, filtered, added to recipient cell medium and incubated for 24 h in the presence of 8 μg/mL polybrene. The next day, 500 μg/mL G418 was added to the medium for selection of the cell populations. For silencing *CTNNAL1* and *KLF5*, lentivirus-based shRNA constructs targeting the luciferase control (TRCN0000072254), *CTNNAL1* (TRCN0000117274 for shCTNNAL1#1 and TRCN0000117275 for shCTNNAL1#2), and *KLF5* (TRCN0000013636 for shKLF5#1 and TRCN0000013637 for shKLF5#2) were obtained from the Taiwan National RNAi Core Facility. HEK293T cells were used as virus-producing cells as mentioned above. The day after infection, 1 μg/mL puromycin was added to the medium for the selection of cell populations.

### Sphere formation assay

For the sphere formation assay, 1000 cells were plated in a 6-well ultralow attachment plate (Corning Glass) and grown in serum-free RPMI medium supplemented with N2 (Invitrogen), 20 ng/ml EGF (PeproTech) and 20 ng/ml bFGF (PeproTech). After 7-14 days of culture, the number of spheres was counted by microscopy.

### Flow cytometric analysis

Cells were suspended in 100 μl of PBS containing 1% FBS and incubated at 4°C for 30 min with PE-conjugated mouse anti-human CD133 (Miltenyi Biotec, 293C3). After washing with PBS, the labeled cells were analyzed by using a FACSCalibur flow cytometer (BD Biosciences).

### ALDEFLUOR assay

The enzymatic activity of aldehyde dehydrogenase (ALDH) was detected by an ALDEFLUOR assay kit (StemCell Technologies, 01700). Cells were suspended in ALDEFLUOR assay buffer containing ALDH substrate (BODIPY-aminoacetaldehyde, BAAA) and incubated for 30 min at 37°C. For the negative control, an aliquot from each sample was treated with 15 μmol/L DEAB (N,N-diethylaminobenzaldehyde, a specific ALDH inhibitor). Using flow cytometry, the percentage of cells expressing high levels of ALDH1 was identified and enumerated. Experiments were performed in triplicate.

### Cytotoxicity assay

For the cytotoxicity assay, 3000 cells were plated in 96-well plates. After adhering overnight, the cells were treated with paclitaxel at a variable concentration, depending on the different cell line, for 72 h. After treatment, cell viability was determined by WST-1 assay (TaKaRa Bio, MK400).

### Coimmunoprecipitation

The harvested cells were lysed in M-PER mammalian protein extraction reagent (Thermo Fisher) at 4 °C. One milligram of cell lysate and 1 μg of the indicated antibody were mixed for 4 h on a rotator at 4 °C. Then, protein A magnetic beads (Millipore) were added to the mixture and incubated for 16 h at 4 °C. The beads were washed and then boiled to release the bound proteins, which were subjected to Western blotting.

### Luciferase reporter assay

The promoter regions of *POU5F1* and *NANOG* were cloned into a pGL3-basic vector (Promega) to generate the luciferase reporter plasmids pGL3-POU5F1_1932_ and pGL3-NANOG_924_. The reporter plasmid plus a pRL-TK-Renilla luciferase internal control vector was transfected alone or in combination with pCMV-Tag2B-α-Catulin and pCMV-Tag2B-KLF5 by using Lipofectamine 2000 transfection reagents (Invitrogen) in accordance with the manufacturer's protocol. One hundred nanograms of pGL3-POU5F1_1932_ or pGL3-NANOG_924_ reporter plasmid (firefly luciferase), 2 ng of internal control plasmid (Renilla luciferase), and 50 ng of α-Catulin or KLF5 plasmid were cotransfected into CL1-0 cells using Lipofectamine 2000. After 24 h of transfection, the cells were lysed, and the luciferase and Renilla activities were measured using a dual-luciferase reporter assay system (Promega) according to the manufacturer's protocol. Firefly luciferase activity was normalized to Renilla luciferase activity for each well of transfected cells.

### *In vitro* and *in vivo* limiting dilution analysis (LDA)

For* in vitro* LDA, cells (1, 10, and 100) were plated in poly(2-hydroxyethyl methacrylate) (poly-HEMA, Sigma)-coated 96-well plates and cultured with sphere-forming medium. After 7-14 days, the wells containing spheres were scored, and the number of positive wells was used to determine the frequency of sphere-forming units with extreme limiting dilution analysis (ELDA) software (http://bioinf.wehi.edu.au/software/elda/index.html) 24.

For *in vivo* LDA, CL1-0 cells (2×10^1^, 2×10^2^, 2×10^3^, 2×10^4^, and 2×10^5^) stably expressing α-Catulin or control plasmids were suspended in 100 μL of Hank's balanced salt solution (HBSS) and subcutaneously injected into the flank region of 8-week-old nonobese diabetic-severe combined immunodeficiency (NOD-SCID) male mice. The injected mice were euthanized after 12 weeks. The number of tumors formed for each injection site was scored to calculate the frequency of lung tumor-initiating cells using ELDA software (http://bioinf.wehi.edu.au/software/elda/index.html) 24.

### Confirmation of interactions by yeast two-hybrid assay

We used a yeast two-hybrid assay to confirm the interaction between α-Catulin and its interacting proteins in *Saccharomyces cerevisiae* strain AH109 as previously described 12.

### Cox proportional hazards model construction and validation

To study the relationships of *CTNNAL1*, *ILK*, and *KLF5* in the overall survival of patients with lung adenocarcinoma, we downloaded a global transcriptome profile containing the mRNA expression of *CTNNAL1*, *ILK*, and *KLF5* and clinical information from Botling's dataset using the Lung Cancer Explorer database (https://lce.biohpc.swmed.edu/lungcancer/) 25. By combining these prognostic gene expression markers with the regression coefficient (β) from a multivariate Cox proportional hazards regression analysis using the coxph() function in the *R* package survival (version 3.1-12), a risk score model was established based on the results of the multivariate Cox regression analysis. Then, the risk score was calculated with the following formula: expression of *CTNNAL1* × β*_CTNNAL1_* + expression of *ILK* × β*_ILK_* + expression of *KLF5* × β*_KLF5_*. According to the median risk score, all patients were assigned to a high-risk or low-risk group. The proportional assumptions used for the Cox proportional hazard model were determined by Kaplan-Meier analysis. Time-dependent receiver operating characteristic (ROC) curves and areas under the curve (AUCs) were plotted by using the *R* package timeROC (version 0.4). In addition, the distribution of the risk scores and survival statuses, along with the gene expression levels of each patient, was analyzed by using R studio.

### Bioinformatics database analysis

Of the data downloaded from the GEO database, the GSE156138 dataset was used to examine *CTNNAL1* expression in lung adenocarcinoma patient-derived tumor spheres and sphere-derived adherent cells, and the GSE40141 dataset was used to analyze α-Catulin-activating stemness-associated genes and signatures. To study the association of α-Catulin and KLF5 at the protein level, we downloaded proteomic and clinical annotated data from the Clinical Proteomic Tumor Analysis Consortium (CPTAC) - lung adenocarcinoma (LUAD) dataset 26.

### Statistical analysis

All data were statistically analyzed using R studio and GraphPad Prism 6.0 software. Two-tailed Student's *t* test was utilized to analyze the difference between the two groups. Pearson's test was applied to determine the correlation between clinicopathological parameters and protein expression. The data are presented as the means ± SD. Differences with *P* < 0.05 were considered significant.

## Results

### α-Catulin promotes the EMT and stemness in NSCLC

**Figure 1:**
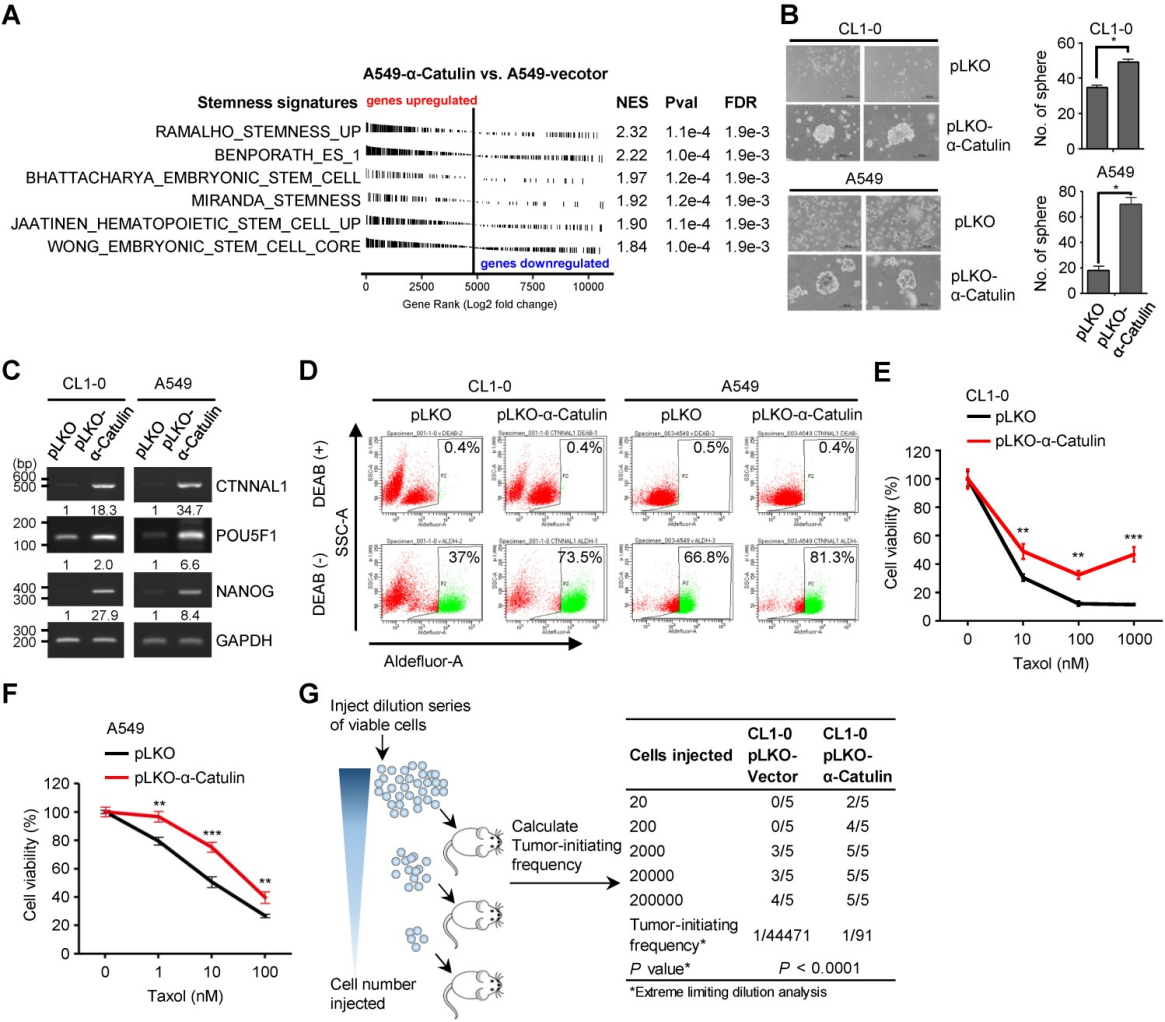
**Overexpression of α-Catulin enhances cancer stemness and tumorigenesis in NSCLC. (A)** GSEA-enrichment plots of the stemness-associated gene sets from microarray data (the GSE40141 dataset) ranked by log_2_ fold change (A549-pLKO-α-Catulin/A549-pLKO-control). NES, normalized enrichment score; Pval, nominal *P*-value; and FDR, false discovery rate. **(B)** Sphere formation assay of CL1-0 and A549 cells with or without α-Catulin overexpression. Formed spheres were photographed (left panel) and quantified (right panel). Scale bar, 200 μm. *P < 0.05 by two-tailed Student *t* test. **(C)** The mRNA expression of *CTNNAL1*, *POU5F1*, and *NANOG* in CL1-0 and A549 cells with or without α-Catulin overexpression. *GAPDH* was used as a loading control. Relative intensities were calculated by ImageJ software and normalized to that of control cells. **(D)** Aldehyde dehydrogenase (ALDH) activity of CL1-0 and A549 cells with or without α-Catulin overexpression. The cells were treated with ALDEFLUOR in the presence or absence of the ALDH inhibitor DEAB (diethylaminobenzaldehyde), and flow cytometry analysis was performed. **(E and F)** CL1-0** (E)** or A549 cells **(F)** with or without overexpression of α-Catulin were treated with different concentrations of paclitaxel for 72 h. Cell viability was measured by WST-1 assay. **P < 0.01 and ***P < 0.001, two-tailed Student's *t* test. **(G)** *In vivo* limiting dilution analysis of CL1-0 cells with or without α-Catulin overexpression. The cells (5 doses, from 2 × 10^1^ to 2 × 10^5^) were subcutaneously injected into NOD-SCID mice. The number of tumors formed was counted to determine the tumor-initiation frequency using ELDA software.

Our previous work demonstrated a role for α-Catulin in promoting NSCLC metastasis 12. In this study, we found that α-Catulin overexpression led to spindle-shaped morphology of the CL1-0 and CL1-5 NSCLC cell lines and induced cells to undergo the EMT (**Figure S1**). Several previous studies had indicated that induction of the EMT generates cells with CSC characteristics 27-31. However, the association between α-Catulin and cancer stemness remains unknown. To investigate the relationship between α-Catulin and stemness, we first compared our global transcriptome profile (based on the GSE40141 data, α-Catulin-overexpressing vs. control A549 cells) to 6 stemness-associated gene sets using a gene set enrichment analysis. All the published stemness-associated gene sets were enriched and exhibited a significant positive correlation with transcripts altered by α-Catulin overexpression (**Figure 1A**). Compared with those in the control cells, most stemness-related genes, such as FGF2, BMI1, and ALDH1A3, were upregulated in the α-Catulin-overexpressing A549 cells (**Figure S2A**). In the clinic, we compared the expression levels between *CTNNAL1* and stemness-associated gene sets obtained from the TCGA-LUAD lung adenocarcinoma dataset. The results also revealed that all of the stemness-associated gene sets were positively correlated with α-Catulin expression (**Figure S2B**). Thus, these observations prompted us to investigate the biological roles of α-Catulin in regulating the stem cell-like properties of NSCLC cells. To investigate the effect of α-Catulin on the stemness of NSCLC cells, CL1-0 and A549 cells were transfected for ectopically expressing the vector control (pLKO) or α-Catulin (pLKO-α-Catulin) (**Figure S3A**). We found that the tumor sphere-forming ability of the α-Catulin-overexpressing CL1-0 cells was increased to approximately 1.5-fold and that of the α-Catulin-overexpressing A549 cells was increased to 3.5-fold (**Figure 1B**). A previous study has shown that coexpression of Oct4 and Nanog induced the EMT, promoted CSC properties and enhanced the metastasis of lung adenocarcinoma 32. Thus, we assessed the effect of α-Catulin overexpression on the expression of stemness-associated transcription factors. Overexpression of α-Catulin in both CL1-0 and A549 cells significantly enhanced both the mRNA expression levels of *POU5F1* and *NANOG* (**Figure 1C**). Lung CSCs have been previously characterized by elevated enzymatic activity of aldehyde dehydrogenase (ALDH) and extracellular expression of CD133 33. To determine the proportion of ALDH-positive NSCLC cells in our study, we performed an ALDEFLUOR assay. Approximately 37% of the CL1-0 cells and 66.8% of the A549 cells showed high ALDH activity (ALDH^high^). Following α-Catulin overexpression, the proportion of ALDH^high^ cells was markedly enriched, to 73.5% in the CL1-0 population and 81.3% in the A549 cell population (**Figure 1D**). Moreover, we found an increased proportion of CD133-positive cells in the α-Catulin-overexpressing CL1-0 cell population compared to that in the control cells (**Figure S3B**). Furthermore, chemotherapy resistance has been reported as one of the important characteristics of CSCs 33. In the present study, we found that α-Catulin-overexpressing CL1-0 cells and A549 cells were more resistant to paclitaxel treatment (**Figure 1E-F**). Next, we performed *in vivo* limiting dilution assays (LDAs) to monitor the effect of α-Catulin on the tumor-initiating capacity of NSCLC cells. Five doses (2×10^1^, 2×10^2^, 2×10^3^, 2×10^4^, and 2×10^5^) of α-Catulin-overexpressing CL1-0 cells and their corresponding control cells were subcutaneously inoculated into NOD/SCID mice. We found that α-Catulin-overexpressing CL1-0 cells displayed higher tumorigenicity than the control cells (**Figure 1G**). Notably, only α-Catulin-overexpressing cells formed visible tumors at doses of 2×10^1^ and 2×10^2^ inoculated cells, suggesting that α-Catulin enhanced the tumor-initiating ability of NSCLC cells (**Figure 1G**). Collectively, these data suggest that α-Catulin enhances the stem-like characteristics of NSCLC cells.

### α-Catulin expression is required to maintain stemness in NSCLC

**Figure 2:**
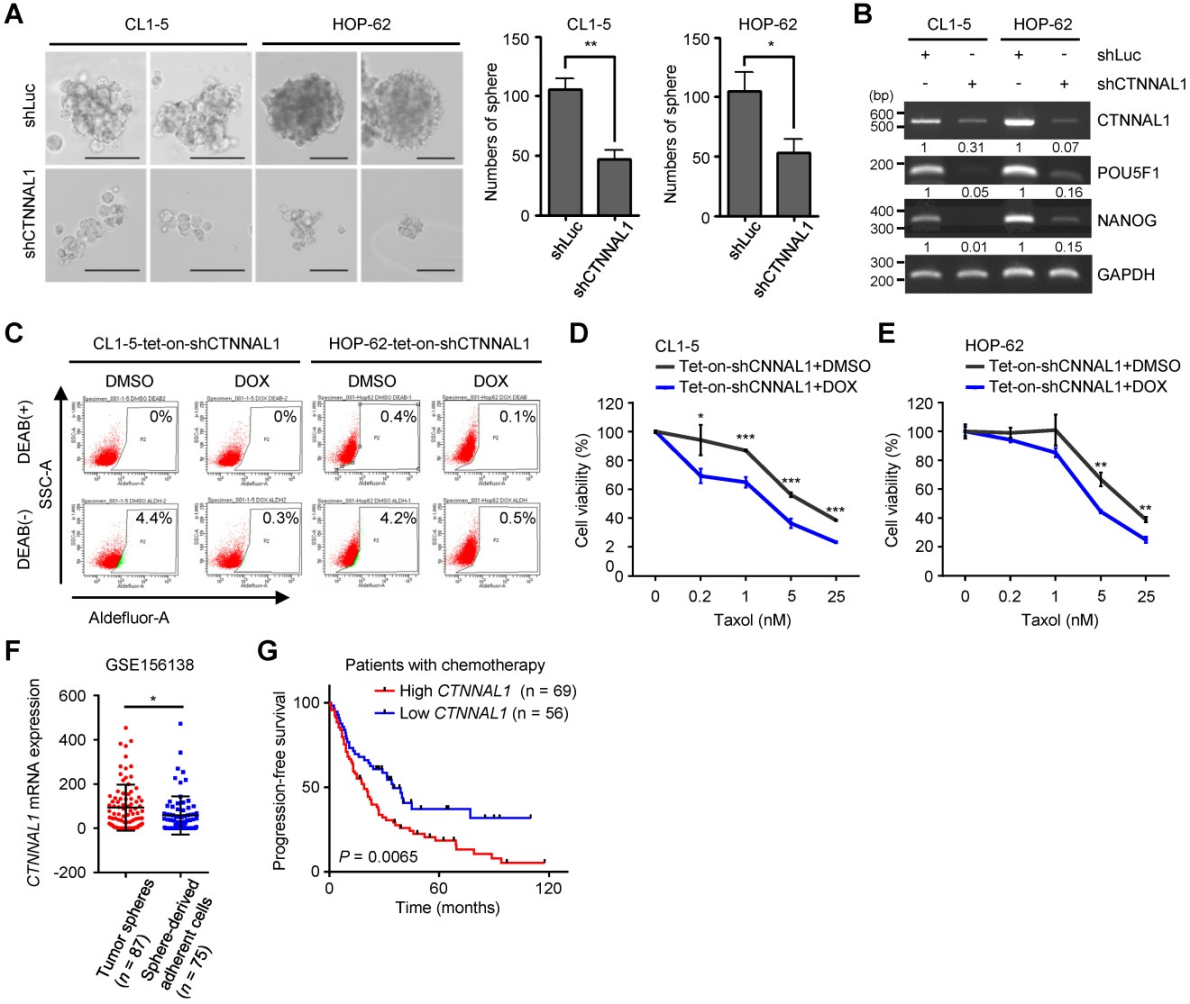
**Silencing of α-Catulin suppresses the characteristics of lung CSCs. (A)** Sphere formation assay of CL1-5 and HOP-62 cells with or without α-Catulin depletion. Formed spheres were photographed and quantified. Scale bar, 100 μm. *P < 0.05 and **P < 0.01 by two-tailed Student's *t* test. **(B)** The mRNA expression of *CTNNAL1*, *POU5F1*, and *NANOG* in CL1-5 and HOP-62 cells with or without α-Catulin depleted. *GAPDH* was used as the loading control. **(C)** Aldehyde dehydrogenase (ALDH) activity of CL1-5 and HOP-62 cells expressing tet-on-shCTNNAL1#2 with or without DOX treatment (1 μg/ml). DMSO, dimethyl sulfoxide and DOX, doxycycline.** (D and E)** CL1-5 **(D)** and HOP-62 **(E)** cells expressing tet-on-shCTNNAL1#2 with or without DOX treatment (1 μg/ml) were treated with different concentrations of paclitaxel for 72 h. The cell viabilities were measured by WST-1 assay. *P < 0.05, **P < 0.01 and ***P < 0.001 by two-tailed Student's *t* test. **(F)** The mRNA expression levels of *CTNNAL1* in lung adenocarcinoma patient-derived tumor spheres and sphere-derived adherent cells were determined by single-cell RNA sequencing using the dataset from Chawla's study 34. *P < 0.05 by two-tailed Student *t* test. **(G)** Association of *CTNNAL1* with progression-free survival in lung cancer patients treated with chemotherapy as determined using Kaplan-Meier Plotter online software. *P*-value was determined by the log-rank test.

To further examine the importance of α-Catulin, we created stable CL1-5 and HOP-62 cell lines expressing either a nontargeting shRNA control or shRNAs to *CTNNAL1*. Both shRNAs to *CTNNAL1* effectively knocked down protein expression. (**Figure S4A**). We found that α-Catulin knockdown suppressed the sphere-forming ability of both CL1-5 and HOP-62 cells (**Figure 2A** and **S4B**). Notably, α-Catulin knockdown inhibited the expression of stemness-associated transcription factors *POU5F1* and *NANOG* in both cell lines (**Figure 2B**). In addition, to further investigate the suppressive effects of α-Catulin knockdown on CSC properties in NSCLC, we established all‐in‐one tetracycline (tet)‐On inducible systems (pAS4.1w.Ppuro-aOn-shCTNNAL1#2) in both CL1-5 and HOP-62 cell lines. Then, we confirmed that the shRNAs effectively led to suppressed protein expression of α-Catulin when the cells were treated with 1 µg/ml doxycycline (DOX) for 2 days to induce the expression of shCTNNAL1#2 (**Figure S4C**). In addition, ALDH-positive cells showed 14-fold and 8-fold decreases in α-Catulin-silenced CL1-5 and HOP-62 cells, respectively (**Figure 2C** and** S4D**). Furthermore, inducible knockdown of α-Catulin in CL1-5 cells resulted in a 2-fold decrease in CD133-positive cells (**Figure S4E**). Moreover, both α-Catulin-silenced CL1-5 and HOP-62 cells were more sensitive to paclitaxel treatment (**Figure 2D-E**). To confirm the expression of α-Catulin in lung CSCs by comparing the results with public datasets, we downloaded the single-cell expression profile for lung cancer patient-derived tumor spheres and sphere-derived adherent cells from Wang's study 34. We found that the expression of *CTNNAL1* was high in the tumor sphere samples but was decreased in the sphere-derived adherent cells generated by continual passage under serum-containing conditions (**Figure 2F**). To investigate the clinical relevance of α-Catulin in the chemotherapeutic response of patients with lung cancer, we used Kaplan-Meier Plotter online software (http://www.kmplot.com) to analyze the progression-free survival data of 125 lung cancer patients treated with chemotherapy 35. We observed that patients with higher *CTNNAL1* had much poorer progression-free survival (*n* = 69; median survival, 19 months) than those showing lower *CTNNAL1* levels (*n* = 56; median survival, 34.8 months), suggesting that expression of α-Catulin correlates with clinical outcomes of chemotherapy in patients with lung cancer (**Figure 2G**). Taken together, these results indicate that α-Catulin expression is required for maintaining CSC properties in NSCLC.

### α-Catulin interacts with KLF5

**Figure 3:**
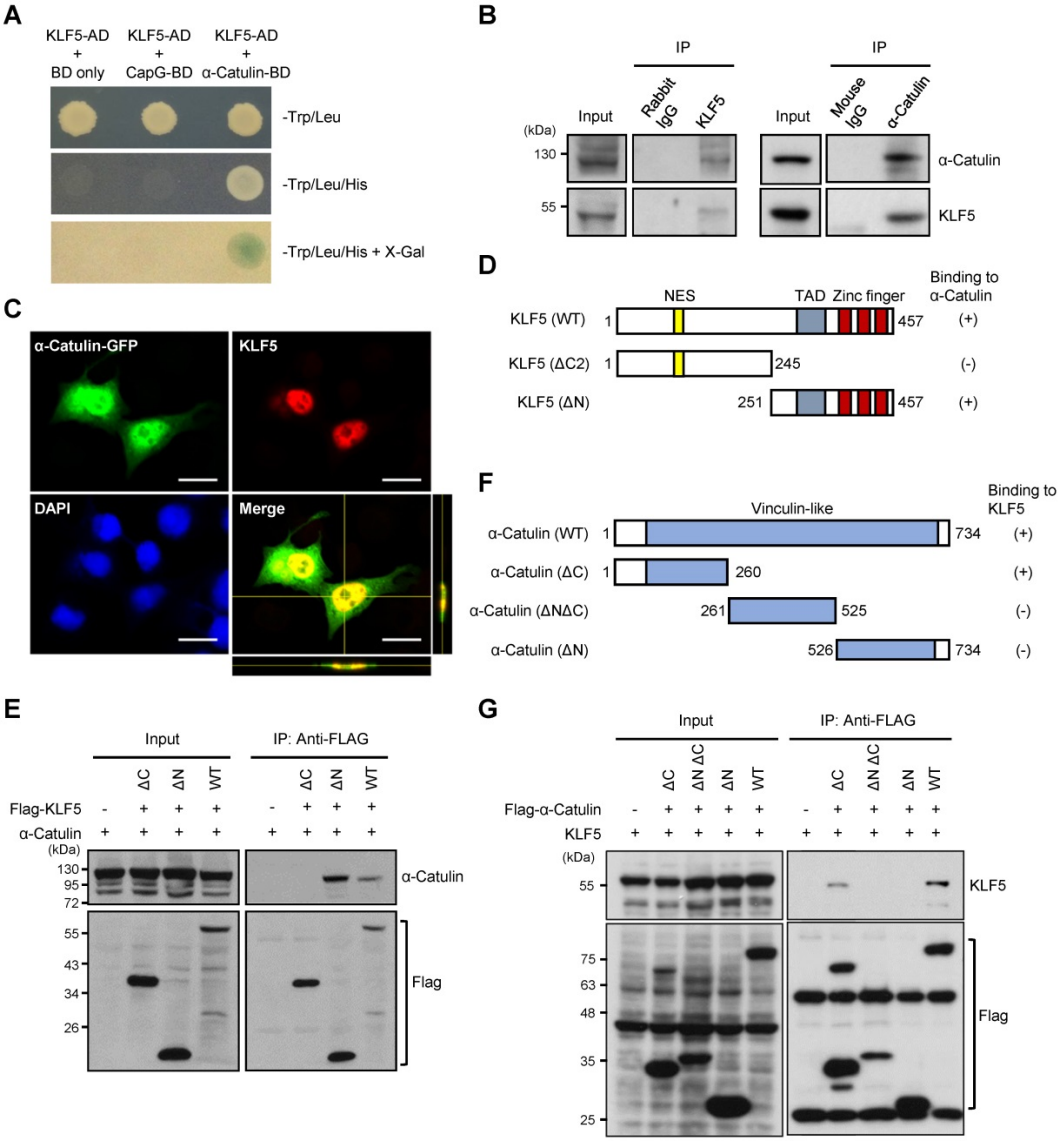
**α-Catulin interacts with the C-terminal region of KLF5. (A)** Yeast two-hybrid analysis of the interaction between KLF5 and α-Catulin. Interactions were confirmed on the basis of cotransformants that are able to grow on histidine-deficient (-His) plates and exhibit β-galactosidase activity. **(B)** Protein lysates from CL1-5 cells were immunoprecipitated with control rabbit IgG, mouse IgG, anti-α-Catulin, or anti-KLF5 antibodies and visualized by immunoblotting with anti-α-Catulin or anti-KLF5 antibodies. **(C)** Immunofluorescence staining and confocal microscopy analyses of KLF5 and α-Catulin of CL1-0 cells co-transfecting with both GFP-α-Catulin and KLF5. Z-stack images were viewed through orthogonal sections. Images indicated the co-localization of α-Catulin and KLF5 in the nucleus (yellow). Scale bar, 20 μm. **(D and F)** Schematic diagrams of full-length KLF5 **(D)** and α-Catulin **(F)** with their truncated mutants. The extent of each interaction is indicated as positive (+) or negative (-). NES, nuclear export signal and TAD, transactivation domain. **(E)** Domain mapping of KLF5 supporting the finding of the α-Catulin-KLF5 interaction. Lysates harvested from CL1-0 cells cotransfected with α-Catulin-GFP and Flag-KLF5 wild-type or truncated mutants were used for immunoprecipitation and immunoblotting analyses with the indicated antibodies. **(G)** Mapping of the α-Catulin required for the α-Catulin-KLF5 interaction. Lysates harvested from CL1-0 cells cotransfected with Flag-α-Catulin wild-type or truncated mutants were used for immunoprecipitation and immunoblotting analyses with the indicated antibodies.

To identify effectors of α-Catulin on CSC-like phenotypes, we performed a yeast two-hybrid (Y2H) screen by using α-Catulin as bait to identify α-Catulin-interacting proteins as previously described 12. Through this screening, we identified several candidates that potentially interact with α-Catulin. Intriguingly, we found that KLF5 stood out as a candidate for interacting with α-Catulin, and it plays a pivotal role in regulating the stemness-associated pathway 36 (**Figure 3A**). Coimmunoprecipitation experiments confirmed the interaction of endogenous α-Catulin and KLF5 proteins in CL1-5 cells (**Figure 3B**). In addition, immunofluorescence staining and confocal microscopy analysis obviously showed a co-localization of KLF5 and α-Catulin in the nucleus of CL1-0 cells co-transfecting with both GFP-α-Catulin and KLF5 (**Figure 3C**). To map the region of KLF5 required for complex formation with α-Catulin, plasmids encoding GFP-tagged full-length α-Catulin and various Flag-tagged KLF5 fragments were cotransfected into CL1-0 cells, coimmunoprecipitated with anti-Flag antibodies, and Western blotted with anti-α-Catulin antibodies (**Figure 3D**). As shown in **Figure 3E**, α-Catulin directly interacted with the C-terminus of KLF5 because deletion of C-terminal residues 246-457 of KLF5 (KLF5-ΔC) led to the mutant inability to bind α-Catulin. In addition, we mapped the region of α-Catulin that interacted with KLF5. Plasmids encoding HA-tagged full-length KLF5 and various Flag-tagged α-Catulin fragments were cotransfected into CL1-0 cells, coimmunoprecipitated with anti-Flag antibodies, and Western blotted with anti-KLF5 antibodies (**Figure 3F**). We found that both α-Catulin-WT and α-Catulin-ΔC (1-260) interacted with KLF5, while α-Catulin-ΔNΔC and α-Catulin-ΔN, which lacked the α-Catulin N-terminus, did not bind to KLF5 (**Figure 3G**). Thus, these results show that the N-terminus of α-Catulin (residues 1-260) and C-terminus of KLF5 (residues 251-457) were important for the formation of the α-Catulin-KLF5 complex.

### KLF5 is important to α-Catulin-driven cancer stemness properties

**Figure 4:**
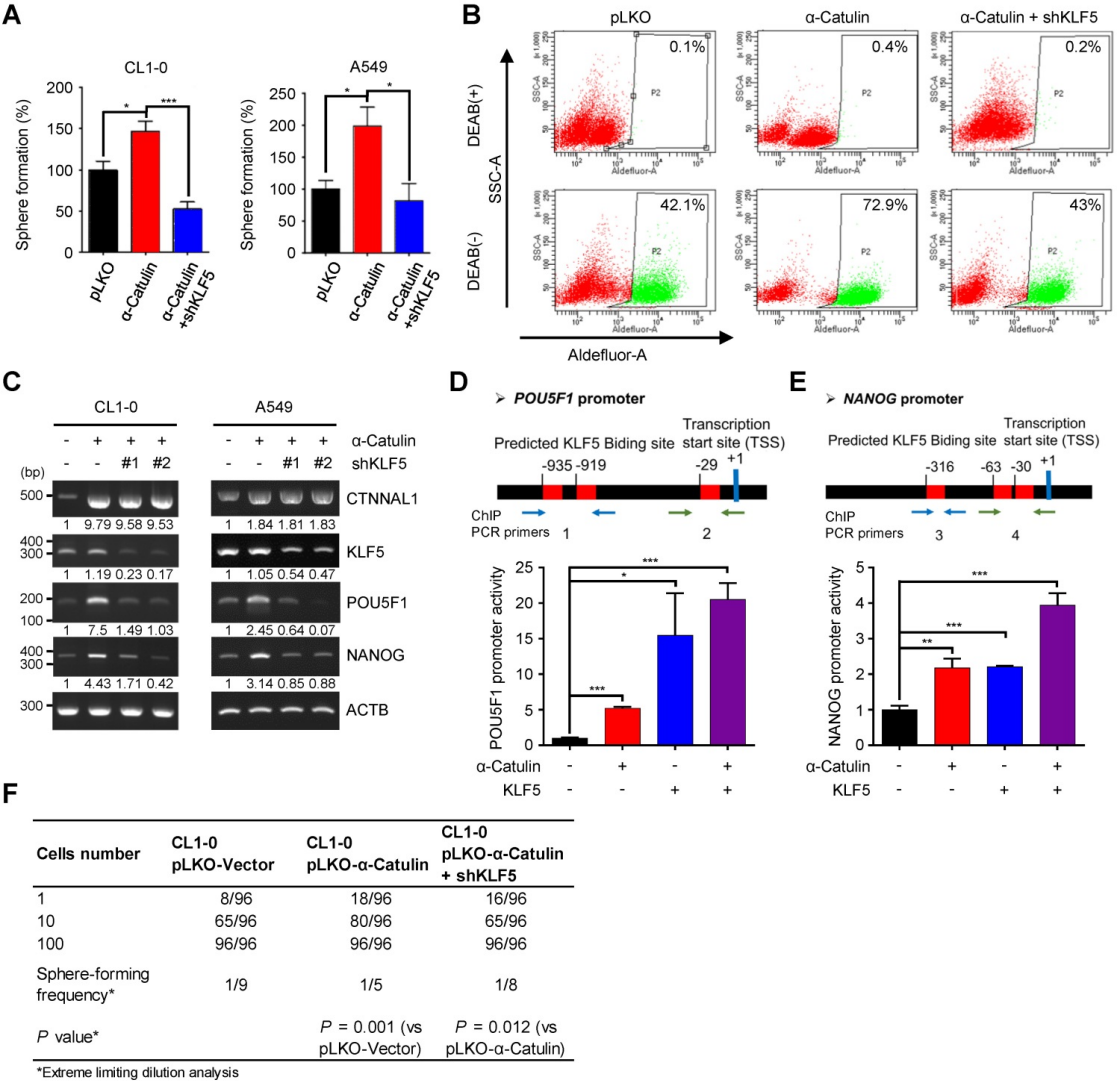
**KLF5 is required for α-Catulin-driven cancer stemness. (A)** Sphere formation assays of CL1-0 and A549 cells expressing α-Catulin or KLF5 shRNA are shown. *P < 0.05 and ***P < 0.001 by two-tailed Student's *t* test. **(B)** ALDH activity of CL1-0 cells expressing α-Catulin or KLF5 shRNA was determined using the ALDEFLUOR assay. **(C)** RT-PCR analysis of CL1-0 and A549 cells expressing α-Catulin-GFP or KLF5 shRNAs. **(D and E)** A diagram of the putative KLF5 DNA binding sites in the promoter regions of *POU5F1* **(D)** and *NANOG* **(E)**. Luciferase activities in CL1-0 cells expressing pCMV-Tag2B-α-Catulin, pCMV-Tag2B-KLF5, and promoter-luciferase reporter constructs (pGL3-POU5F1_1932_ and pGL3-NANOG_924_) were determined. Reporter activities were normalized to that of Renilla luciferase activity. *P < 0.05, **P < 0.01, and ***P < 0.001 by two-tailed Student's *t* test. **(F)** The frequency of sphere-forming cells expressing α-Catulin or KLF5 shRNA was measured by* in vitro* limiting dilution analysis. Sphere-containing wells were counted to calculate the frequency of sphere-forming units using ELDA software.

To determine whether KLF5 was the direct functional mediator of α-Catulin-driven stem cell-like properties in NSCLC, we synthesized short-hairpin RNAs (shRNAs) for KLF5 and transduced these shRNAs into CL1-0 and A549 cells overexpressing α-Catulin. Overexpression of α-Catulin in the CL1-0 and A549 cells resulted in approximately 1.5-fold and 2-fold increases in sphere formation, respectively, whereas knockdown of KLF5 abolished these effects (**Figure 4A**). In addition, knockdown of KLF5 in α-Catulin-overexpressing cells diminished α-Catulin-induced ALDH activity (**Figure 4B**). Moreover, knockdown of KLF5 in α-Catulin-overexpressing cells reduced α-Catulin-induced expression of the stemness factors *POU5F1* and *NANOG* (**Figure 4C**). Previous studies have shown that KLF5 promoted the transcription of *POU5F1* and *NANOG* by directly binding to promoter regions 18. An *in silico* analysis was used to predict the putative KLF5-binding sites in the promoter regions of *POU5F1* and *NANOG*. To investigate the transcriptional regulation of *POU5F1* and *NANOG*, we inserted promoter regions of human *POU5F1*, -1452 ~ +480, and *NANOG*, -58 ~ -981, into the pGL3-basic vector to generate reporter plasmids of pGL3-POU5F1_1932_ and pGL3-NANOG_924_. We transfected either pGL3-POU5F1_1932_ or pGL3-NANOG_924_ together with pRL-TK and pCMV-Tag2B-KLF5 and pCMV-Tag2B-α-Catulin plasmids into cells and then measured luciferase activity using a dual luciferase assay. Compared with the pLKO control, overexpression of α-Catulin and KLF5 increased the luciferase activity of pGL3-POU5F1_1932_ by almost 5-fold and 15-fold, respectively (**Figure 4D**). Similarly, we observed that overexpression of either α-Catulin or KLF5 increased the luciferase activity of pGL3-NANOG_924_ by approximately 2-fold (**Figure 4E**). Intriguingly, coexpression of α-Catulin and KLF5 further enhanced the highest promoter activities of pGL3-POU5F1_1932_ and pGL3-NANOG_924_ (**Figure 4D-E**). Immunofluorescence staining and chromatin immunoprecipitation assays showed that α-Catulin and KLF5 interact with each other in nucleus and at the endogenous *POU5F1* and *NANOG* promoters (**Figure 3C and S5**). These results suggest that α-Catulin cooperates with KLF5 to enhance the transcription of *POU5F1* and *NANOG*. Using an *in vitro* LDA, we found that knockdown of KLF5 in α-Catulin-overexpressing CL1-0 cells abolished the α-Catulin-induced sphere formation frequency (**Figure 4F**). Taken together, these results suggest that α-Catulin-induced CSC phenotypes were mediated by KLF5.

### α-Catulin protects KLF5 from WWP1-mediated proteasomal degradation

**Figure 5:**
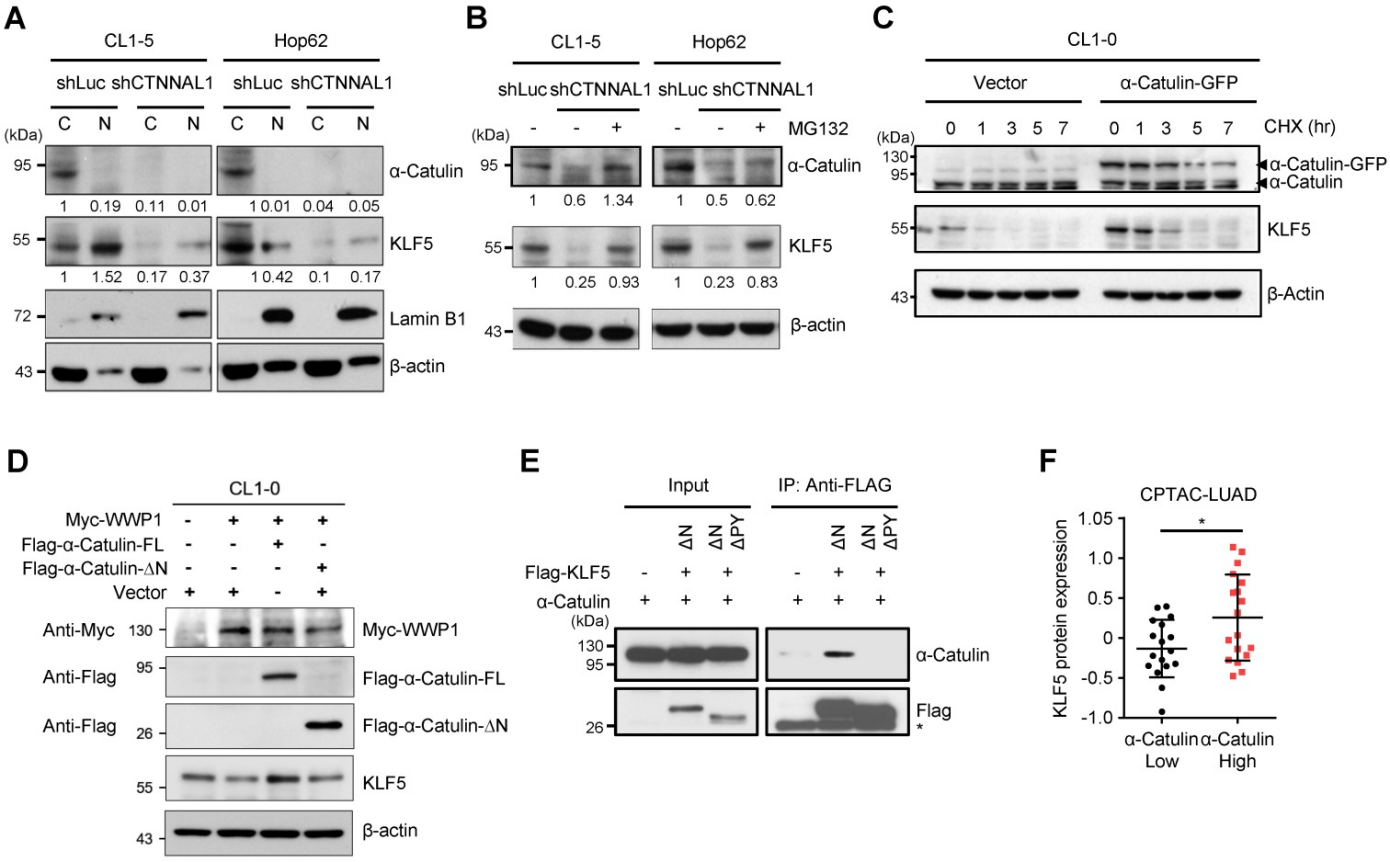
**α-Catulin protects KLF5 from WWP1-mediated proteasomal degradation. (A)** Western blot analysis of α-Catulin, KLF5, Lamin B1 (nuclear marker) and β-actin in cytoplasmic (C) and nuclear (N) fractions of CL1-5 and HOP-62 cells with or without α-Catulin depleted. **(B)** α-Catulin-depleted and control CL1-5 and HOP-62 cells were treated with or without MG132 (10 μM, 24 h). Western blot analysis was performed with the indicated antibodies. **(C)** CL1-0 cells with or without α-Catulin-GFP overexpression were treated with CHX (100 μg/ml) for different periods of time. **(D)** Western blot analysis of KLF5 in CL1-0 cells cotransfected with WWP1 and full length or N-terminal truncated α-Catulin.** (E)** Lysates harvested from CL1-0 cells cotransfected with α-Catulin-GFP and Flag-KLF5 truncated mutants, C-terminus of KLF5 with PY motif (KLF5-ΔN) or without PY motif (KLF5-ΔNΔPY), were used for immunoprecipitation and immunoblotting analyses with the indicated antibodies.** (F)** Protein expression of KLF5 in high-α-Catulin-expressing (-0.38~0.57) and low-α-Catulin-expressing (-1.18~-0.39) tumors in patients with grade 3 lung adenocarcinoma as recorded in the CPTAC-LUAD datasets. *P < 0.05 by two-tailed Student *t* test.

Previous studies have shown that WWP1, an E3 ubiquitin-protein ligase, interacted with KLF5 by binding the PY motif, a destruction motif in the KLF5 transactivation domain, and caused the ubiquitination and proteasomal degradation of KLF5 37. Our results showed that α-Catulin interacted with the C-terminal region of KLF5 containing the PY motif. However, the influence of α-Catulin on the protein stability of KLF5 remains unclear. To assess its effect, we first knocked down α-Catulin, which caused a robust reduction in both cytoplasmic and nuclear KLF5 expression in CL1-5 and HOP-62 cells (**Figure 5A** and** S6A**). Further investigation showed that the reduction of KLF5 by α-Catulin knockdown was blocked by the proteasome inhibitor MG132 (**Figure 5B** and **S6B**). Furthermore, a cycloheximide (CHX) chase analysis showed that α-Catulin overexpression increased the protein half-life of KLF5 (**Figure 5C**). To further elucidate the contribution of WWP1 to α-Catulin-mediated effects, α-Catulin and WWP1 were cotransfected into CL1-0 cells. Compared with the control, α-Catulin overexpression decreased WWP1-mediated KLF5 protein degradation, while N-terminal-truncated α-Catulin without KLF5 binding capability did not have such effect. (**Figure 5D**). Coimmunoprecipitation experiments further proved that α-Catulin binds to KLF5 through the PY motif of KLF5 (**Figure 5E**). To investigate the association between the protein expression of α-Catulin and KLF5 in clinical specimens, we downloaded proteomic data of poorly differentiated lung adenocarcinomas from the CPTAC dataset (*n* = 35) 26. Notably, the protein expression of KLF5 was significantly increased in tumors with high expression of α-Catulin (*n* = 18) compared to tumors with low expression of α-Catulin (*n* = 17) (**Figure 5F**). Taken together, these data suggest that α-Catulin enhanced the protein stability of KLF5 by blocking WWP1-mediated proteasomal degradation of KLF5.

### The small-molecule inhibitor OSU-T315 disrupts α-Catulin-ILK-KLF5 complex formation and suppresses cancer stemness in NSCLC

**Figure 6:**
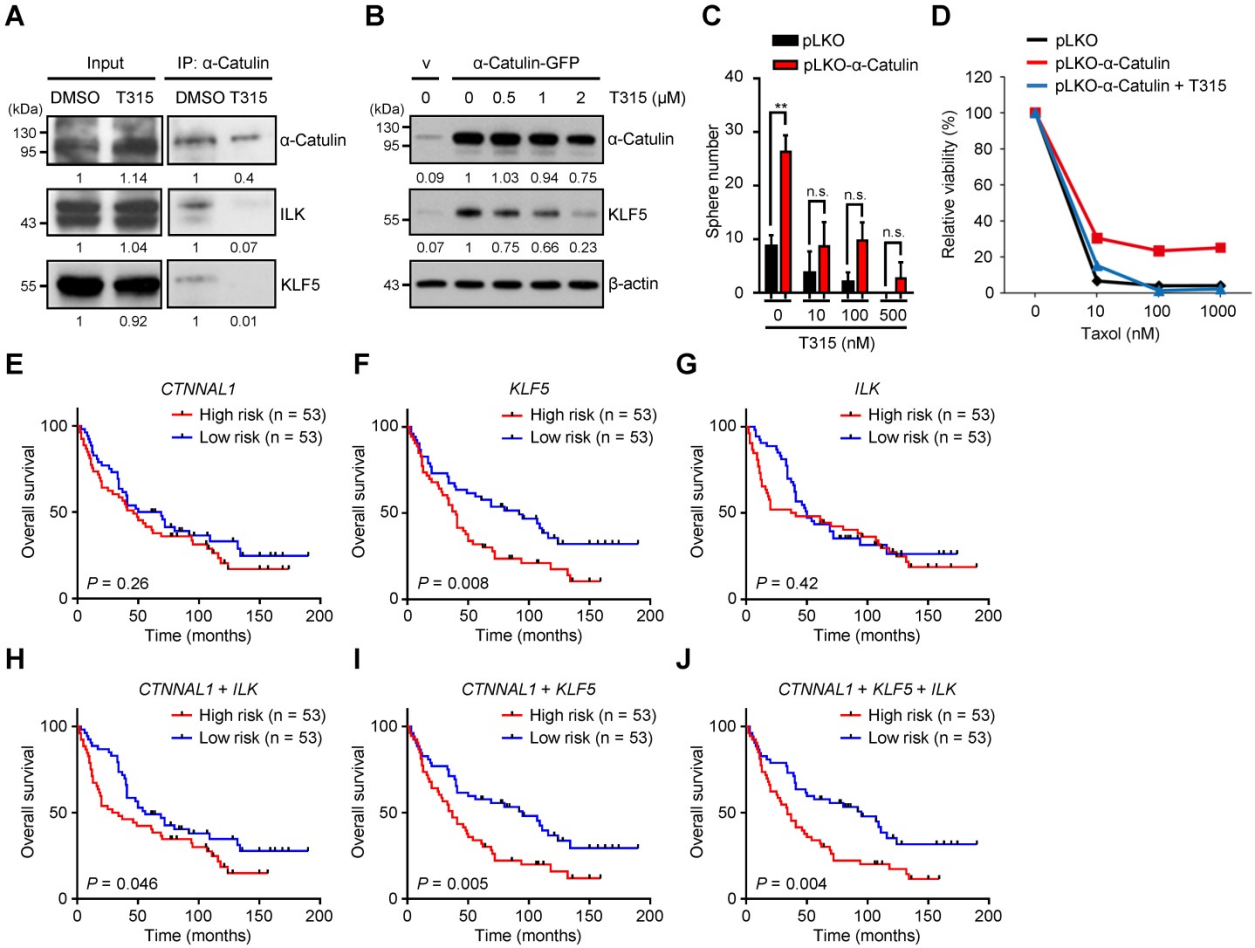
**Treatment with the ILK inhibitor OSU-T315 suppresses α-Catulin-induced cancer stemness, and the *CTNNAL1*/*ILK*/*KLF5* 3-gene signature predicts poor overall survival in the NSCLC cohort. (A)** α-Catulin was immunoprecipitated from CL1-5 cells with or without 2 μM OSU-T315 treatment. Western blot analysis was performed with the indicated antibodies. **(B)** Western blot analysis of CL1-0 cells with or without α-Catulin overexpression treated with the indicated concentrations of OSU-T315. β-Actin was used as the loading control in both cell groups. **(C)** CL1-0 cells with or without α-Catulin overexpression were treated with or without the indicated concentrations of OSU-T315, and sphere formation assays were performed. **P < 0.01 by two-tailed Student *t* test. **(D)** Control and α-Catulin-overexpressing CL1-0 cells were treated with OSU-T315 and the indicated concentrations of paclitaxel. Viable cells were identified by WST-1 assay. **(E-J)** Survival analysis of 106 patients with lung adenocarcinoma as recorded in Botling's NSCLC cohort and performed on the basis of *CTNNAL1* **(E)**, *KLF5* **(F)**, and* ILK* **(G)** gene expression using the Kaplan-Meier method. Kaplan-Meier analyses of overall survival based on patient data stratified into high- and low-scoring groups using the gene signature of *CTNNAL1* plus *ILK* **(H)**, *CTNNAL1* plus *KLF5* **(I)**, and *CTNNAL1* plus *ILK* plus *KLF5* **(J)**. *P*-values were determined by log-rank test.

We have previously found that α-Catulin interacted with ILK, induced ILK activation, and produced a positive signaling feedback loop that contributed to cancer metastasis 12. Targeting ILK by OSU-T315, an ILK inhibitor, efficiently inhibited the activation of downstream genes 12. Here, we confirmed that α-Catulin, ILK, and KLF5 were able to form a complex that could be disrupted by OSU-T315 (**Figure 6A**). Furthermore, the protein stability of KLF5 enhanced by α-Catulin was inhibited by OSU-T315 treatment (**Figure 6B**). OSU-T315 does not reduce the mRNA levels of *CTNNAL1* and *KLF5* (**Figure S7A**). OSU-T315-reduced protein stability of KLF5 could be blocked by the proteasome inhibitor MG132 (**Figure S7B**). Moreover, we observed that a dominant-negative kinase-dead mutant of ILK (A262V) decreased the levels of KLF5 in CL1-0 cells (**Figure S7C**). These data suggest that the enzyme activity of ILK is important for the KLF5 protein stability. OSU-T315 treatment significantly suppressed both tumor sphere formation and paclitaxel resistance driven by α-Catulin overexpression (**Figure 6C-D**). Overexpression of KLF5 partially rescues the OSU-T315-suppressed cancer sphere formation (**Figure S7D**). In summary, these results indicate that the ILK inhibitor OSU-T315 effectively targeted α-Catulin-mediated cancer stemness in NSCLC.

### *The CTNNAL1*/*ILK*/*KLF5* three-gene signature predicts a poor outcome for patients with NSCLC

To investigate the clinical relevance of *CTNNAL1*, *ILK*, and *KLF5* in NSCLC patients' overall survival, we downloaded the clinical characteristics of 106 patients with lung adenocarcinoma from Botling's dataset 38. Among these factors, only *KLF5* was a prognostic marker in the cohort (hazard ratio [HR] = 1.87, log-rank *P* = 0.008); *CTNNAL1* was not a prognostic marker (HR = 1.3, log-rank *P* = 0.26) or *ILK* (HR = 1.21, log-rank *P* = 0.42) (**Figure 6E-G**). However, the *CTNNAL1* plus *ILK* 2-gene signature could predict poor overall survival of NSCLC patients (hazard ratio [HR] = 1.6, log-rank *P* = 0.046) (**Figure 6H**). This result is consistent with our previous analysis 12. Interestingly, we found that the *CTNNAL1* plus *KLF5* 2-gene signature could distinguish NSCLC patients with poor survival better than either the *CTNNAL1*-only or *KLF5*-only signature (hazard ratio [HR] = 1.94, log-rank *P* = 0.005) (**Figure 6I**). The Kaplan-Meier survival curves showed that NSCLC patients with the worst survival rate in the group exhibited high levels of *CTNNAL1*/*ILK*/*KLF5* (hazard ratio [HR] = 1.97, log-rank *P* = 0.004) (**Figure 6J**). Next, the prognostic value of a 3-gene signature was assessed based on time-dependent ROC curves. The AUCs of the 3-year and 5-year risk scores were 0.65 (**Figure S8**), which proved that the 3-gene signature had a high specificity and sensitivity for predicting the overall survival of NSCLC patients. Taken together, our results suggest that the 3-gene signature of *CTNNAL1* plus *ILK* plus *KLF5* can be used to predict the outcome of patients with lung adenocarcinoma.

## Discussion

**Figure 7:**
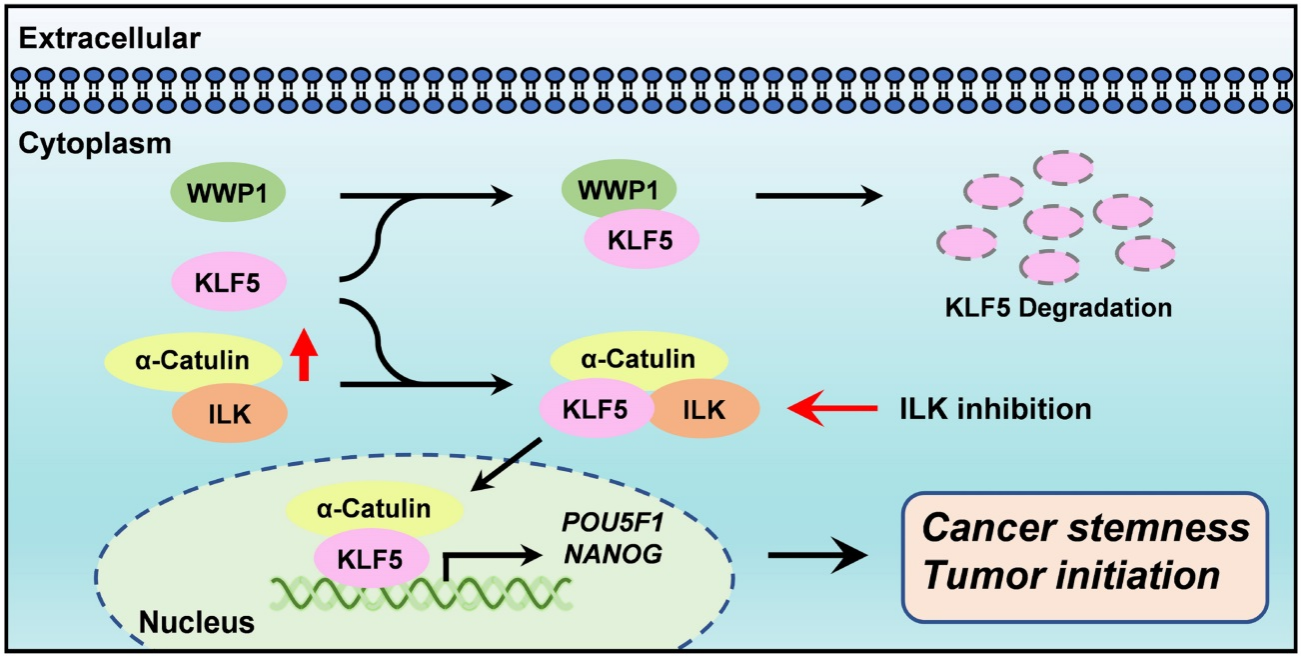
**Diagram summarizes how α-Catulin enhances cancer stemness and therapeutic targeting of lung CSCs by ILK inhibitors.** α-Catulin enhances CSC properties by interacting with KLF5, which protects KLF5 from WWP1-mediated degradation. Targeting α-Catulin-regulated signaling by the ILK inhibitor OSU-T315 disrupted the α-Catulin-KLF5 interaction and promoted the degradation of KLF5, indicating therapeutic potential for suppressing lung CSCs.

α-Catulin has been linked to tumor progression, invasion and metastasis 9-12. However, the exact function of α-Catulin in the regulation of cancer stemness remains unknown. We demonstrated here that α-Catulin overexpression enhanced the measures of stem cell markers, such as ALDH activity and CD133 expression, and increased the expression of pluripotency transcription factors, including Oct4 and Nanog. Notably, high expression of α-Catulin was detected in lung tumor spheres derived from patients but not in sphere-derived adherent cells. Analysis of yeast two-hybrid screening and coimmunoprecipitation in our study revealed that KLF5, which is an important stemness driver, physically interacted with α-Catulin. Mechanistically, α-Catulin interacted with the C-terminal domain of KLF, which contains an PY motif that binds the E3 ubiquitin ligase WWP1. α-Catulin overexpression blocked WWP1-mediated KLF5 degradation. Moreover, treatment with an ILK inhibitor disrupted the interaction of α-Catulin and KLF5 and α-Catulin-driven sphere formation. Taken together, this study provides a rationale for targeting the α-Catulin-ILK-KLF5 complex to suppress cancer stemness in NSCLC (**Figure 7**).

In this study, we found that inhibition of ILK by OSU-T315 treatment caused disruption of α-Catulin-ILK-KLF5 protein complex formation, while KLF5 was relatively labile. In one mechanism of OSU-T315, the C-terminus of KLF5 containing the PY motif does not bind α-Catulin to prevent WWP1-mediated proteasome degradation. In addition, Ser303 phosphorylation of KLF5 by GSK3-β has been previously shown to promote the degradation of KLF5 by enhancing the interaction of KLF5 and the E3 ubiquitin ligase Fbw7 39. A different study has shown that ILK induced the phosphorylation of Akt-Ser473 and GSK-3β-Ser9, which inactivated GSK-3β in various cell types 40. However, in our previous work, we have demonstrated that α-Catulin cooperated with ILK to promote the phosphorylation of Akt, but not GSK-3β, in NSCLC cell lines 12. Therefore, treatment with ILK inhibitors greatly inhibited the phosphorylation of Akt at Ser473 12. Given that Akt activation has been shown to inhibit the serine phosphorylation of KLF5 41, we speculated that suppression of α-Catulin-induced KLF5 stabilization by the ILK inhibitor may also through the inactivation of Akt signaling. Thus, our study provides a new finding linking ILK activity to KLF5 signaling.

The work presented here indicates that KLF5 plays an important role in α-Catulin-driven cancer stemness. KLF5 overexpression has been previously identified in a wide variety of cancers, including lung cancer, cervical cancer, thyroid cancer, pancreatic cancer, and melanoma 42-46. In addition, KLF5 plays a pivotal role in maintaining CSCs in several cancers, such as ovarian cancer, liver cancer, and breast cancer 19, 47, 48. Our findings indicate that KLF5 is highly associated with poor overall survival of patients with lung adenocarcinoma, suggesting that targeting KLF5-driven malignancy may provide an opportunity for developing lung cancer therapeutics (**Figure 6F**). This result agrees with a previous study by Zhang and colleagues showing that KLF5 is overexpressed and correlated with poor survival in patients with primary NSCLC 42. Our analyses of human lung adenocarcinoma specimens from the CPTAC dataset revealed higher expression of the KLF5 protein in high-α-Catulin-expressing tumors than in low-α-Catulin-expressing tumors. Importantly, we discovered a *CTNNAL1*/*ILK*/*KLF5* signature that was highly associated with poor prognosis in NSCLC patients. Therefore, our findings reveal an instrumental mechanism underlying the stabilization and activation of KLF5 signaling.

Ample evidence has previously indicated that CSCs are important for tumor growth and resistance to chemotherapeutic agents 49. Considering that CSCs share many similar biological properties with normal stem cells, targeting CSCs may also affect normal stem cells 50. Thus, the identification of new drugs that selectively target CSCs but not normal stem cells is greatly needed. Previous studies have shown that Oct4 and Nanog can enhance both tumor-initiating capability and the EMT, and the expression of either Oct4 or Nanog has been correlated with a poor outcome for patients with lung adenocarcinoma 32. In this study, we found that α-Catulin enhanced the stabilization of KLF5 to increase the transcription of *POU5F1* and *NANOG*, suggesting an important role for α-Catulin in controlling the expression of stemness-associated transcription factors **(Figure 4C-D)**. Establishing the critical role of the α-Catulin-KLF5 complex in promoting cancer cell stemness and resistance to chemotherapy may indicate that this complex is a molecular target for the therapy of human malignancies. Given that inhibiting KLF5 offers a potentially important therapeutic strategy to target CSCs 17, 51, the current study provides a new strategy that targets the α-Catulin-KLF5 complex to suppress CSCs in NSCLC.

## Supplementary Material

Supplementary figures.
