# Supplementary material for: α-Catulin promotes cancer stemness by antagonizing WWP1-mediated KLF5 degradation in lung cancer
Source: Theranostics. 2022 Jan 1;12(3):1173–86. doi: 10.7150/thno.63627 (PMC8771551; doi:10.7150/thno.63627)

Supplementary Materials for Manuscript Entitled

α-Catulin promotes cancer stemness by antagonizing WWP1-mediated KLF5 degradation in lung cancer

Chia-Hao Tung[1], Meng-Fan Huang[1], Chen-Hsien Liang[2], Yi-Ying Wu[3], Jia-En Wu[1], Cheng-Lung Hsu[4], Yuh-Ling Chen[2,5,#] and Tse-Ming Hong [1,3,#]

[1]Institute of Clinical Medicine, College of Medicine, National Cheng Kung University, Tainan, Taiwan.

[2]Institute of Basic Medical Sciences, College of Medicine, National Cheng Kung University, Tainan, Taiwan.

[3]Clinical Medicine Research Center, National Cheng Kung University Hospital, College of Medicine, National Cheng Kung University, Tainan, Taiwan.

[4]Division of Hematology-Oncology, Department of Internal Medicine, Chang Gung Memorial Hospital, Chang Gung University, Taoyuan, Taiwan.

[5]Institute of Oral Medicine, College of Medicine, National Cheng Kung University, Tainan, Taiwan.

Running title: α-Catulin promotes lung cancer stemness

Supplementary figure legends

Figure S1. α-Catulin promotes EMT in NSCLC cells. (A) Representive images of CL1-0-pLKO and pLKO-α-Catulin cells. Scale bar, 50 μm. **(B)** Protein expression of α-Catulin and EMT markers in CL1-0 cells with or without α-Catulin overexpression. **(C)** Representive images of CL1-5-shLuc and shCTNNAL1#2 cells. Scale bar, 100 μm. **(D)** Protein expression of α-Catulin and EMT markers in CL1-5 cells with or without *CTNNAL1* silencing.

Figure S2. α-Catulin expression is positively associated with stemness-associated signatures and genes. (A) The expression levels of genes related to cancer stemness in our previous microarray analysis (GSE40141). The data were presented as $\log_2$ ratios of the normalized expression in A549-pLKO-α-Catulin to the A549-pLKO-control cells. **(B)** GSEA-enrichment plots of the stemness-associated gene sets in TCGA lung adenocarcinomas ($n = 509$) ranked by Pearson correlation to *CTNNAL1* expression. NES, normalized enrichment score; Pval, nominal *P*-value; FDR, false discovery rate.

Figure S3. Overexpression of α-Catulin enhances the proportion of CD133 positive cells in NSCLC cells. (A) Protein expression of α-Catulin in CL1-0 cells with or without α-Catulin overexpression. β-acitn was used as a loading control. Relative protein intensities were calculated by Image J software and normalized to control cells. **(B)** Flow cytometry analysis of CD133 positive cells in CL1-0 cells with or without α-Catulin overexpression. ***$P < 0.001$ by two-tailed Student *t* test.

Figure S4. Silencing of *CTNNAL1* suppresses the characteristics of lung CSCs. (A) Protein

expression of α-Catulin in CL1-5 and HOP-62 cells with or without α-Catulin depletion. β-actin was used as loading control. **(B)** Sphere formation assay of CL1-5 and HOP-62 cells with or without α-Catulin depletion. Formed spheres were photographed and quantified. Scale bar, 100 μm. **(C)** Protein expression of α-Catulin in CL1-5 and HOP-62 cells expressing tet-on-shCTNNAL1#2 with or without treatment of DOX (1 μg/ml). β-acitn was used as a loading control. Relative protein intensities were calculated by ImageJ software and normalized to control cells. DOX, doxycycline; DMSO: Dimethyl sulfoxide. **(D)** Aldehyde dehydrogenase (ALDH) activity of CL1-5 and HOP-62 cells with or without α-Catulin depletion. **(E)** Flow cytometry analysis of CD133 positive cells in CL1-5 cells expressing tet-on-shCTNNAL1#2 with or without treatment of DOX (1 μg/ml). $^{**}P < 0.01$ and $^{***}P < 0.001$ by two-tailed Student's t test.

Figure S5. α-Catulin and KLF5 co-expression increases the binding of KLF5 to the *POU5F1* promoter. (A) The scheme showed the predicted KLF5 DNA binding sites and the regions which amplified by ChIP primers 1 to 4 in the promoters of *POU5F1* and *NANOG*. **(B)** CL1-0 cells were overexpressed α-Catulin and KLF5. ChIP assay was performed with anti-KLF5 antibody, and the precipitated DNA was amplified with primers 1 to 4, respectively. NTC, non-template control.

Figure S6. The reduction of KLF5 by α-Catulin knockdown could be blocked by the proteasome inhibitor MG132. (A) Western blot analysis of α-Catulin, KLF5, Lamin B1 (nuclear marker) and β-actin in cytoplasmic (C) and nuclear (N) fractions of CL1-5 and HOP-62 cells with or without α-Catulin depleted. **(B)** α-Catulin-depleted and control CL1-5 and

HOP-62 cells were treated with or without MG132 (10 μM, 24 h). Western blot analysis was performed with the indicated antibodies.

Figure S7. Overexpression of KLF5 partially rescues the OSU-T315-suppressed cancer stemness. (A) The qRT-PCR analysis of *CTNNAL1* and *KLF5* shows that OSU-T315 does not reduce the mRNA levels of these two genes. **(B)** OSU-T315-reduced protein stability of KLF5 could be blocked by the proteasome inhibitor MG132. **(C)** Overexpression of kinase-dead ILK mutant (ILK-A262V) suppressed the expression of KLF5 in CL1-0 cells. **(D)** Overexpression of KLF5 partially rescues the OSU-T315-suppressed cancer sphere formation.

Figure S8. Time-dependent receiver operating characteristic (ROC) curve analysis for the *CTNNAL1/ILK/KLF5* 3-gene signature in lung adenocarcinoma. AUC, area under the curve.

Supplementary Figure S1

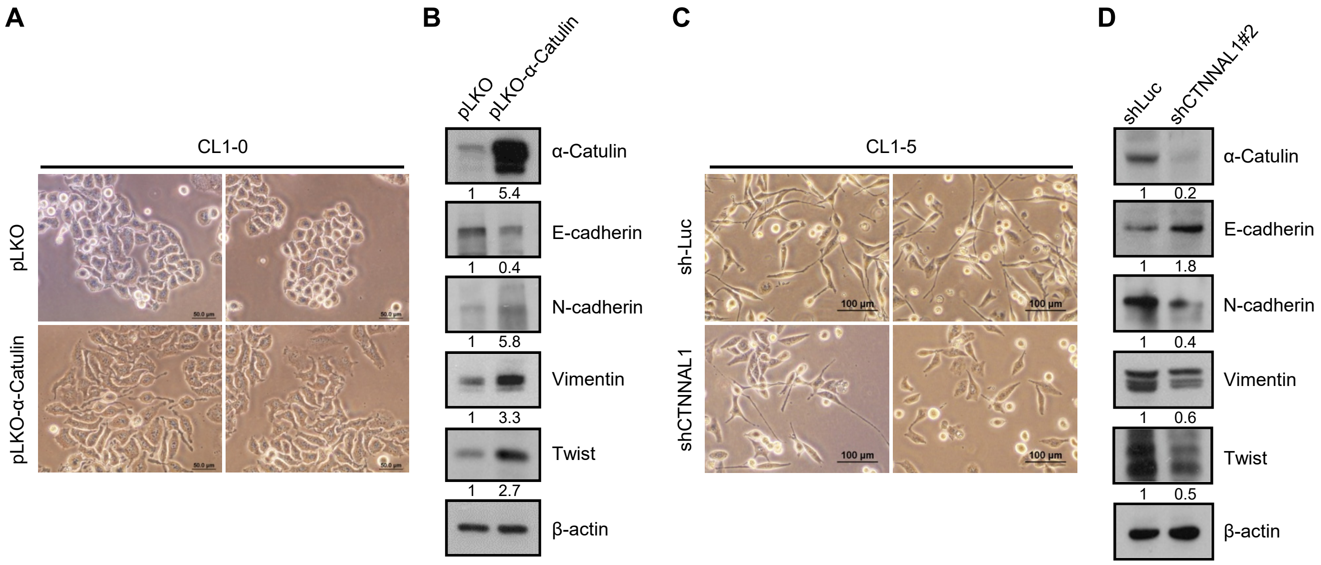

Supplementary Figure S2

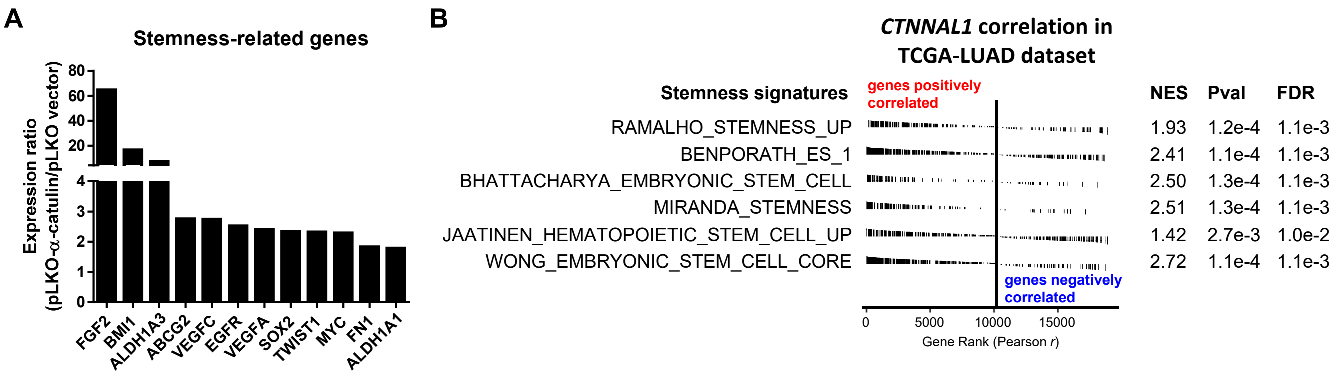

Supplementary Figure S3

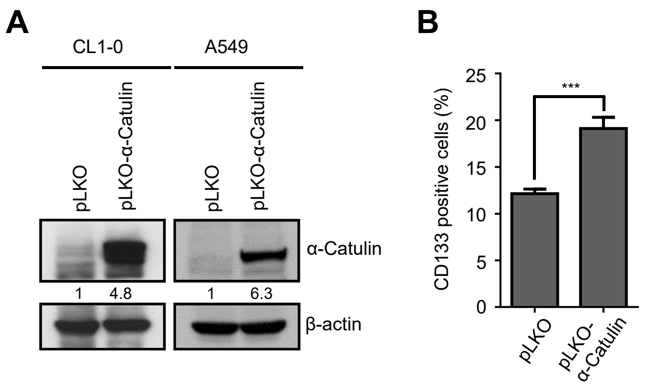

Supplementary Figure S4

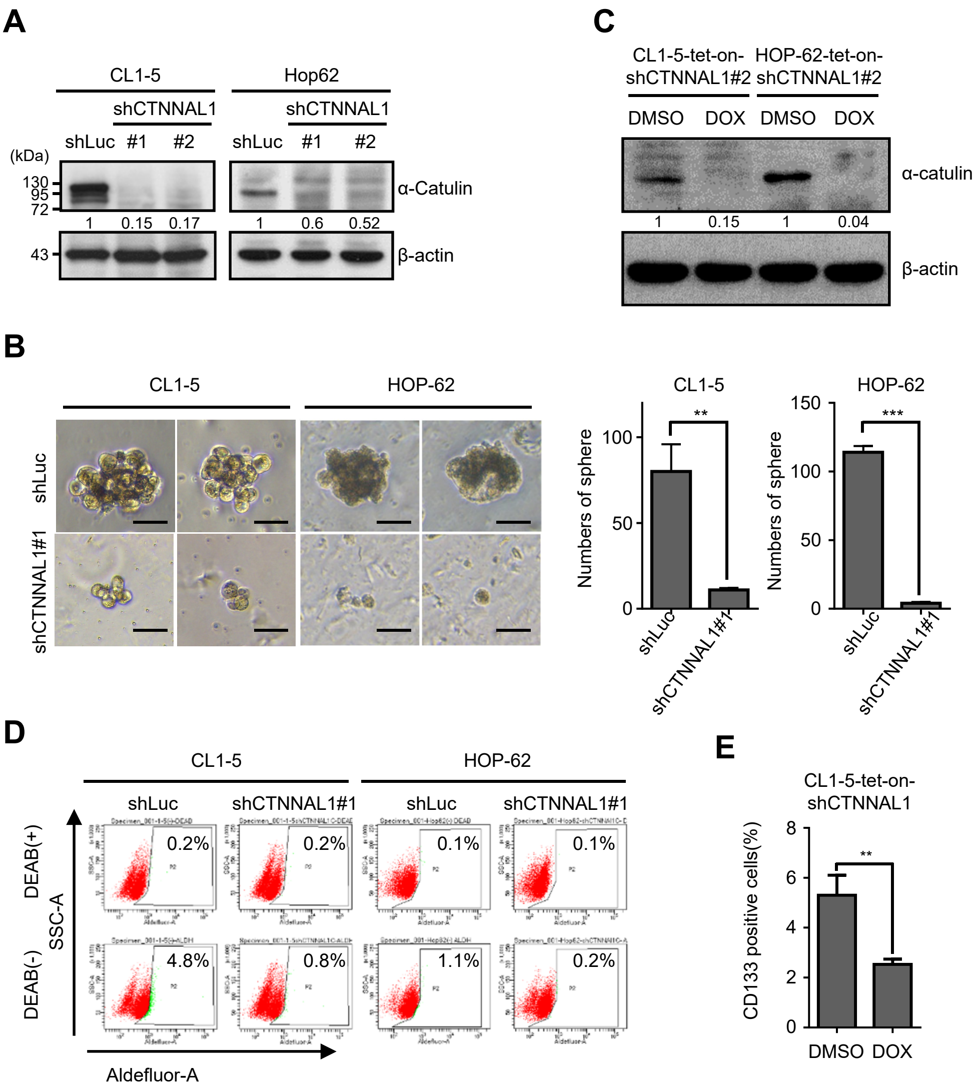

Supplementary Figure S5

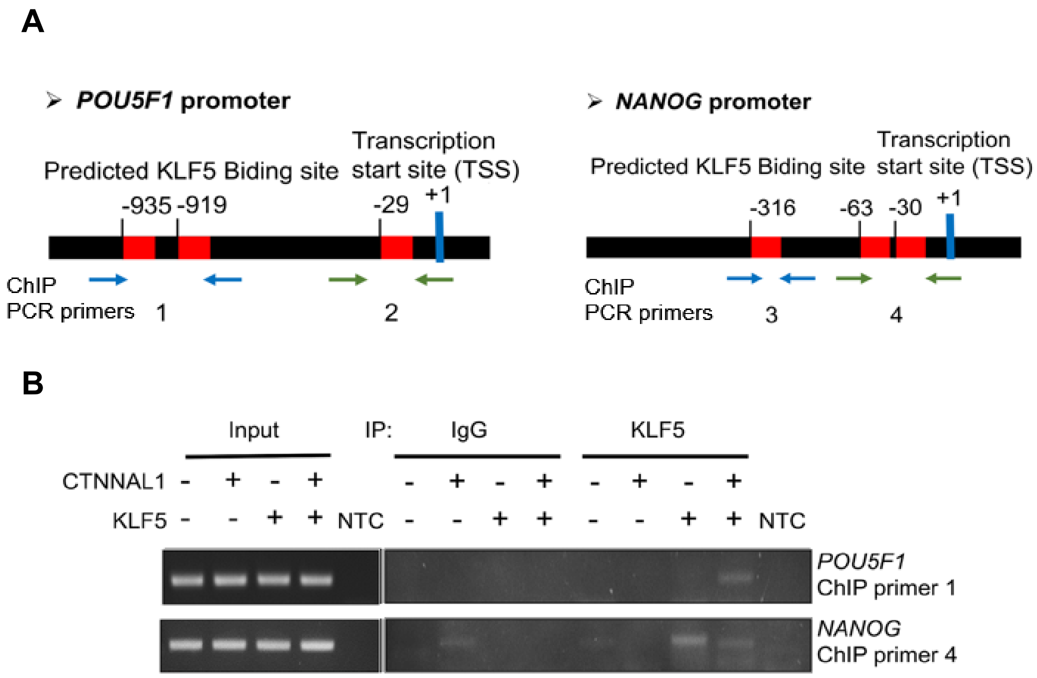

Supplementary Figure S6

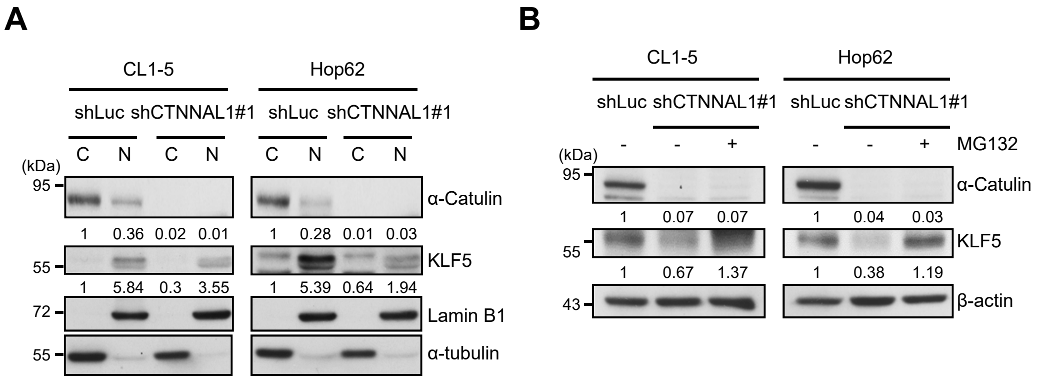

Supplementary Figure S7

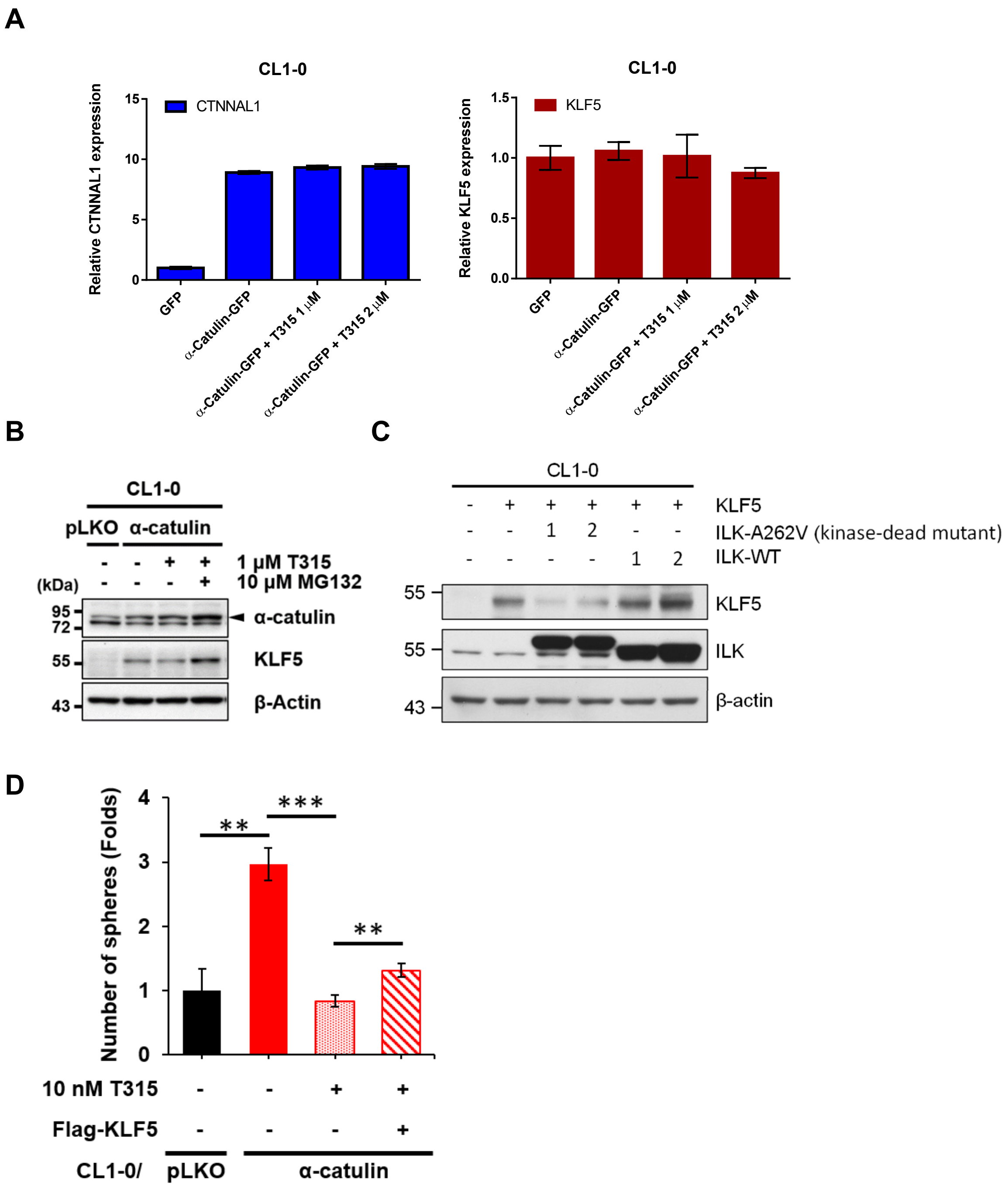

Supplementary Figure S8

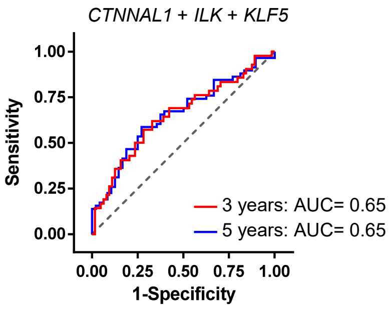

Supplement: Supplementary file 1 — Supplementary figures. [file thnov12p1173s1.pdf]
